# Bacteria

**Published:** 1893-05

**Authors:** N. S. Hoff


### ARTICLE V.

### BACTERIA.

BY DR. N. S. HOFF.

Putrefaction is always accompanied by the presence of bacteria; and bacteriologists maintain that this process cannot take place without the presence of micro-organisms. It has been reasonably demonstrated that micro-organisms are the definite cause of specific diseases. The cholera bacillus has been successfully isolated, and the disease produced in healthy animals by inoculation. This is also true of many others; anthrax typhoid fever, consumption, etc., can be produced by inoculating healthy animals with pure specimens of the microorganisms found in these diseases. It is the confident expectation of bacteriologists that characteristic bacteria of every known disease will be determined by further research. Just how these minute organisms produce such destructive changes in living tissue is not definitely known. It has been observed that conditions favorable to their growth are moisture, body temperature, and a favorable medium. Excessive temperature, hot or cold, will inhibit their growth, or destroy them. 105 degrees F. will inhibit most varieties; at 32 degrees F. they will not grow. They will not grow in either an acid or alkaline medium. It is evident that the process is very closely allied if not identical with fermentation. It has been found that there is present in tissue affected with pathogenic bacteria a peculiar nitrogenous waste product, which has evidently been produced by the activity of the micro-organisms. It very closely resembles vegetable alkaloids, which are formed in putrefying mixture, and is usually poisonous. It is called a ptomain (from *ptoma*—a corpse) because it was first isolated from dead bodies. It is the ptomain that produces in animals the characteristic disease, poisonous or fatal results.

There exist in the body at all times various and numerous species or forms of bacteria. It is a wise provision of nature that it is so, for we find that the presence of the non-pathogenic orders have a decided tendency to modify or correct the action of the disease-producing species. The favorable conditions and presence of germs in so great a variety would seem to promise the speedy overthrow of all vital function in the tissues of the body, were it not for the fact that nature has made provision for resisting the encroachment and interference of these organisms, which is brought about principally in three ways:

*First*, the fluids of the body are capable of destroying them by their acid character, or prevent their growth by their alkaline reaction.

*Second*. the white corpuscles of the blood and the connective tissue cells have the power to destroy the bacteria by taking them into their interior and digesting them. This can only be accomplished to a definite extent, and when more bacteria are present than the cells can digest, the cells themselves give way and are destroyed by the bacteria, and we have death and the putrefactive process set up.

*In the first place*, the bacteria overcome and destroy each other or are destroyed by their own products. If a wound is inoculated with several kinds of bacteria, one species will gain the ascendency at the expense of the others, till it has by destruction so contaminated the media in which it is operating by its own activity and has produced a condition in which it cannot grow; an excess of acid, or alkali, or alcohol; then its activity ceases and the germ becomes incompetent to produce its species. At this stage another kind of germ that thrives on the conditions present may take up the fight and change conditions to his own hurt or disadvantage, and he in turn is compelled to give way to a more vigorous successor.

This kind of warfare will continue indefinitely, or till by surgical and medical interference or renewed and reinforced vital function, the system is enabled to overcome the parasitic influence and re-establish a normal and vigorous functional condition.

It is at this point that the interference of the surgeon or physician is needed to turn the process in favor of the attacked structures.

That the disinfecting process may be intelligently accomplished it is not necessary that we recognize the peculiar variety of micro-organisms which may be present, as fortunately the larger number will succumb to comparatively harmless agencies, and all can be reached by drugs contained in our materia medica.

The conditions produced by the different species are varied, according to the germ, but those which are especially concerned in the formation of gases and odors are of particular interest to the dentist. But all forms are liable to produce both gases and odors under the conditions present in most dental lesions, such as putrefaction of the pulp, alveolar abscess, pyorrhœa alveolaris. It is not important here to enumerate the particular action of special species or forms.

We must classify all agents or drugs which may be used to overcome the influence or results of the action of micro-organisms under the general head of antizymotics, that is, agents which will prevent fermentation, for we are not justified in separating fermentation from putrefaction, for fermentation is an essential feature of putrefaction even in animal structures.

In the clinical application of antizymotics for the correction of disease, we find that all drugs and methods to be efficient must be applied in such concentration or power to effect the desired results, that continued application would result in the destruction of large amounts of valuable tissue that is not all or only slightly affected by the encroachment of zymotic influences. And we have learned also that tissue which is not inoculated, or only slightly affected, can be kept in an aseptic condition by attenuated solutions of strong germicides or by milder agents, which we will designate antiseptics. We will, therefore, make a sub-classification of antizymotics into disinfectants and antiseptics. The disinfectant is the means whereby we aim to remove all infectious matter and agencies from the tissue; the antiseptic is the means used to preserve the cleanliness obtained by the use of the disinfectant.—*Dental Register*.

